# Prediction of the mortality rate in the intensive care unit for early sepsis patients with combined hypoalbuminemia based on machine learning

**DOI:** 10.1097/MD.0000000000043610

**Published:** 2025-08-01

**Authors:** ZhanJin Wang, XuXia A, Lei Liu, Ying Zhou, FuYuan Li, ZhangTuo Xue, JunJie Cai, Kaihao Du, Zhan Wang

**Affiliations:** a Qinghai University, XiNing, China; b Affiliated Hospital of Qinghai University, XiNing, China; c Department of Medical Engineering Integration and Translational Application, Affiliated Hospital of Qinghai University, XiNing, China.

**Keywords:** hypoalbuminemia, machine learning, predictive model, sepsis

## Abstract

This study aims to predict the mortality rate among septic patients with early-onset hypoalbuminemia in the intensive care unit (ICU) using machine learning algorithms. Utilizing patient data from the MIMIC-IV and eICU databases, we divided MIMIC-IV samples into training and internal validation sets, with eICU samples serving as an external validation set. We developed the predictive model using various feature selection techniques and machine learning algorithms, and evaluated its performance using metrics such as AUC, accuracy, precision, recall, and F1 score. The SHAP method was used for model interpretability. The CatBoost model, developed using recursive feature elimination, outperformed other algorithms, demonstrating robust generalization with AUC values of 0.845, 0.746, and 0.827 across the respective datasets. This pioneering study presents a machine learning model with high accuracy and robust extrapolation capabilities for predicting mortality rates in septic patients with early-onset hypoalbuminemia in the ICU, providing valuable decision support for clinicians.

## 1. Introduction

Sepsis is a global health concern, affecting millions annually, with an adult hospital incidence rate ranging from 133 to 267 per 100,000 person-years and a mortality rate of 22.9% to 30.7%.^[1,2]^ Particularly in developing and low-income countries, the incidence and mortality of sepsis are notably higher, imposing significant economic and medical burdens.^[3]^ Despite a recent decrease in sepsis incidence, it remains a substantial challenge for healthcare systems worldwide.^[1,4]^ This condition is characterized by a dysregulated host response to infection, leading to organ dysfunction.^[5]^ Progression of sepsis exacerbates capillary permeability, causing albumin redistribution outside the vasculature and resulting in hypoalbuminemia.^[6]^ Research indicates that severe hypoalbuminemia complicating sepsis often leads to multiple organ failure, heightening mortality risk and culminating in adverse outcomes.^[7]^

When serum albumin levels fall below 3.5 g/dL, the condition can be diagnosed as hypoalbuminemia.^[8]^ Research indicates that with every 1 g/dL decrease in serum albumin concentration, there is an 89% increase in incidence and a 137% rise in mortality rate.^[9]^ Additionally, hypoalbuminemia can disrupt the pharmacokinetics of antibiotics, potentially leading to treatment failure or excessive drug toxicity.^[10]^ Given these insights, accurately predicting the early mortality rate of septic patients with hypoalbuminemia is paramount for timely intervention. Such interventions could facilitate the improvement of patients’ protein levels and subsequently enhance their prognosis. Consequently, we aim to develop a machine learning predictive model to anticipate the overall mortality rate of septic patients exhibiting early-onset hypoalbuminemia in the intensive care unit (ICU), with the goal of guiding early interventions based on these forecasts.

The utilization of machine learning in sepsis complication and prognosis analysis has become widespread in recent years, with the development of diverse predictive models facilitating clinical decision-making.^[11]^ For instance, Yue et al devised a predictive model employing the XGBoost algorithm, effectively anticipating acute kidney injury occurrence in septic patients, achieving an impressive the area under the ROC curve (AUC) value of 0.817.^[12]^ Additionally, Zhou et al study focuses on identifying high-risk factors associated with all-cause mortality in septic patients with acute kidney injury.^[13]^ Dou et al proposed an early prediction model for sepsis, surpassing manual scoring systems like SOFA and significantly aiding clinicians in early sepsis detection.^[14]^ Zhuang et al research centers on predicting the probability of postoperative sepsis, vital for early patient evaluation and mitigating postoperative sepsis risk.^[15]^ Overall, machine learning offers potent tools for enhancing patient prognosis, mitigating mortality risk, and easing disease burden.

This study endeavors to develop a machine learning model to forecast the early mortality rate among septic patients concurrently experiencing hypoalbuminemia in the ICU. The envisaged model aims to aid clinicians in evaluating the mortality hazard associated with septic patients exhibiting hypoalbuminemia, discerning those at high risk, and facilitating prompt intervention and tailored therapeutic strategies. To enhance comprehension of the model’s predictive foundation, the study employs the shapley additive explanations (SHAP) method to elucidate the interconnection between risk factors and predictive outcomes.

## 2. Method

### 2.1. Data source

The data for this study were sourced from 2 databases, namely MIMIC-IV (v2.2) and eICU (v2.0), to conduct a retrospective cohort study focusing on septic patients. MIMIC-IV serves as a substantial single-center publicly available database containing comprehensive clinical data of patients admitted to the Beth Israel Deaconess Medical Center from 2008 to 2019.^[16]^ On the other hand, the eICU database comprises detailed clinical information of patients treated ICU across over 200 hospitals in the United States from 2014 to 2015. This encompasses demographic data, vital signs, laboratory test results, microbiology culture results, imaging data, treatment processes, medication records, and survival information.^[17]^ For model development and internal validation, we utilized sepsis data from MIMIC-IV, while data from eICU were employed for external validation. It is pertinent to note that health information obtained from the MIMIC-IV database has undergone de-identification, thereby obviating the need for patient consent. The author (ZhanJin Wang) has been granted permission to extract data from the database for research purposes, as indicated by certification number 59,293,246. Furthermore, this study has received approval from the Institutional Review Board of the Massachusetts Institute of Technology.

**Figure 1.**
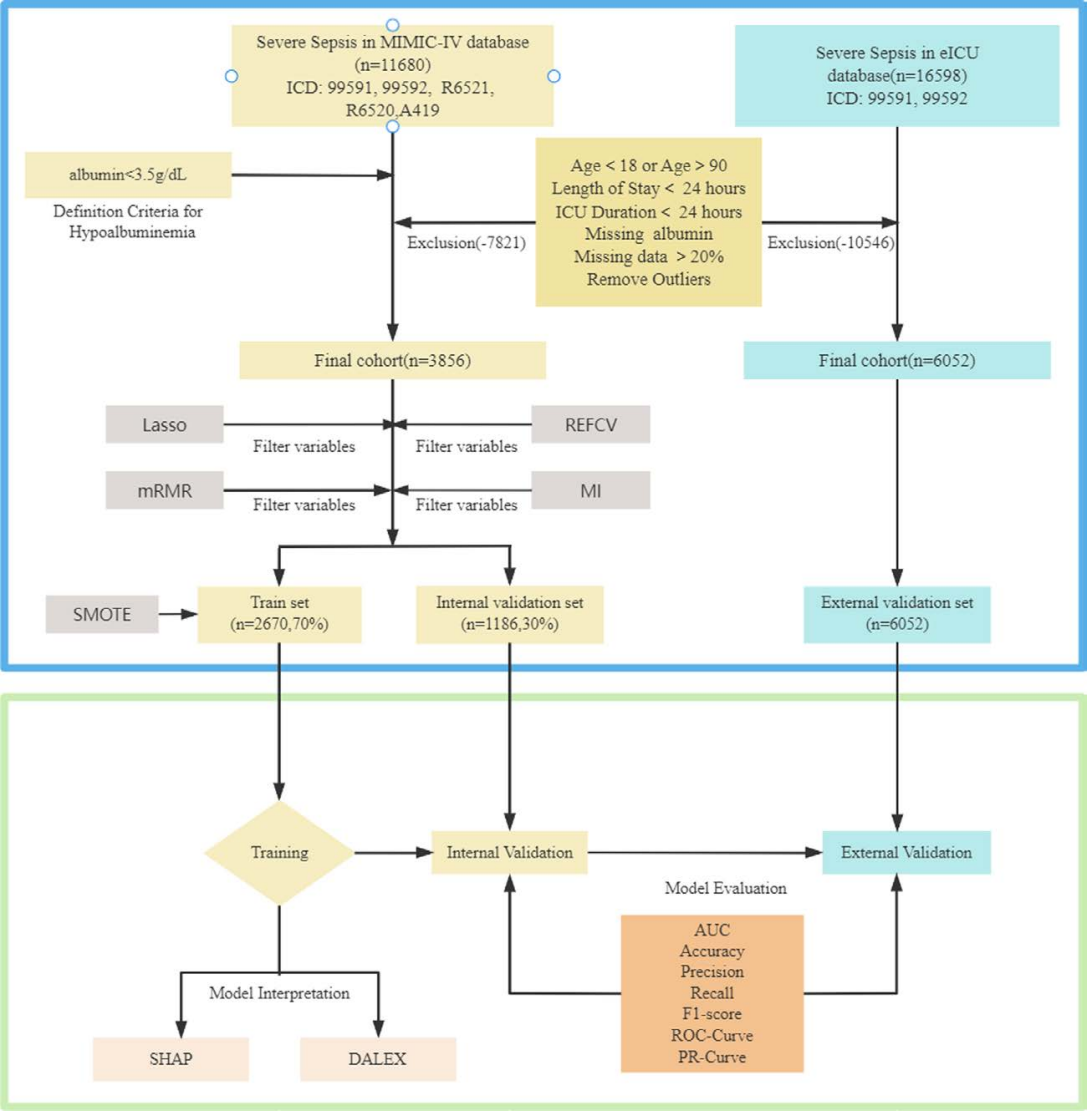
Complete research process diagram.

In the process of data extraction, we utilized the SQL language within Navicat Premium (version 15.2.7.0). Within the MIMIC-IV database, sepsis patients were identified utilizing ICD-9 and ICD-10 disease codes 99592, A419, 99591, R6521, and R6520. In the eICU database, patient identification was conducted using ICD-9 codes 99592 and 99591. Furthermore, individuals presenting with serum albumin levels below 3.5 g/dL upon admission to the ICU were classified as having hypoalbuminemia. In order to ensure data integrity and research precision, stringent exclusion criteria were implemented. Initially, individuals below the age of 18 or above 89 were excluded to mitigate bias stemming from their distinct physiological and pathological circumstances. Subsequently, patients with hospital stays surpassing 28 days or ICU stays shorter than 24 hours were omitted to mitigate the impact of treatment interventions and care duration on the dataset. Moreover, patients with a single ICU admission were excluded to ensure comprehensive representation of ICU patient characteristics. Lastly, data sets exhibiting more than 20% missing values were eliminated to minimize potential adverse effects on the predictive capability of the model. The inclusion criteria for the eICU dataset mirrored those aforementioned. These methodological steps are depicted in Figure 1.

### 2.2. Variable selection

The primary aim of this study is to predict the mortality of septic patients with hypoalbuminemia during their ICU stay. We extracted the following predictive indicators from both the MIMIC-IV and eICU datasets: demographic data, encompassing age, gender, and race (Caucasian, African American, Asian, and others). Initial vital signs post-admission, including heart rate, systolic and diastolic blood pressure, mean arterial pressure, respiratory rate, temperature, and oxygen saturation. Initial laboratory findings following ICU admission, covering parameters such as anion gap, aspartate aminotransferase, alkaline phosphatase, alanine aminotransferase, eosinophils, basophils, lymphocytes, neutrophils, monocytes, bicarbonate, bilirubin, sodium, potassium, calcium, chloride, magnesium, creatinine, hematocrit, hemoglobin, glucose, platelets, international normalized ratio, prothrombin time, activated partial thromboplastin time, blood urea nitrogen, white blood cells, red blood cells, lactate dehydrogenase, and mean corpuscular volume. Historical medical records regarding cardiovascular diseases, chronic pulmonary conditions, and acute renal failure. Early administration of vasopressors, mechanical ventilation, and renal replacement therapy post-ICU admission. Glasgow Coma Scale (GCS) assessment and urine output on the initial day.

### 2.3. Statistical analysis

In this research, we performed data processing and analysis using R software (version 4.3.1). Initially, we evaluated the normal distribution of continuous variables using normality tests. Continuous variables conforming to a normal distribution were depicted as mean ± standard deviation, while those deviating from normality were represented using the median and interquartile range (IQR). Categorical variables were articulated in terms of counts and percentages. We utilized t-tests to compare normally distributed continuous variables, whereas non-normally distributed ones were assessed using the Kruskal–Wallis test. The comparison of categorical variables was conducted using either the χ² test or Fisher exact test. All analyses were executed using the autoReg package (version 0.3.3) within R (4.3.1). In this study, results with a *P*-value below .05 were deemed statistically significant.

### 2.4. Data preprocessing and feature engineering

In this study, we developed a machine learning predictive model for the mortality rate of critically ill septic patients with hypoproteinemia using Python (version 3.11) and R (version 4.3.1). The dataset was obtained from the MIMIC-IV database and underwent deduplication as part of the data cleaning process. During the initial screening of the MIMIC and eICU databases, we retained only features with missing data not exceeding 20% (Figs. S1 and S2, Supplemental Digital Content, https://links.lww.com/MD/P548). In order to tackle the problem of missing data, we employed the “miceforest” package (version 5.6.3) within the Python environment. Subsequently, we filled in the missing data through the application of the multiple imputation method based on Random Forest Imputer. The MIMIC-IV dataset was randomly divided into training and internal validation sets in a 7:3 ratio. Given the dataset’s imbalance, we applied the synthetic minority over-sampling technique (SMOTE) for data handling. The over-sampling technique, SMOTE, was exclusively conducted within the training dataset. The specific parameters for SMOTE were set as follows: with k_neighbors = 5, m_neighbors = 10, svm_estimator, random_state = 42. Before commencing the model training process, both standardization and normalization procedures were performed on all feature variables. In the case of categorical variables such as gender and ethnicity, the Label Encoding method was utilized to assign corresponding integer values, facilitating their integration into the subsequent analytical framework. For continuous variables, including those related to vital signs and laboratory test outcomes, given that they manifested non-normal distribution characteristics, the RobustScaler approach was uniformly adopted. To elaborate, initially, the median and interquartile range of each individual feature were computed. Subsequently, relying on these statistical metrics, the data points within each feature were transformed accordingly. Thereafter, through the application of the fit_transform method, the parameters governing the feature distribution within the training data were learned, thus successfully achieving the standardized transformation of the training data and ensuring that the feature variables were confined within a suitable scale range, which is conducive to the subsequent model training and performance optimization. To improve model accuracy and generalization, this study employed 4 different feature selection methods: Least Absolute Shrinkage and Selection Operator (Lasso), Mutual Information (MI), Maximum Relevance Minimum Redundancy (mMRM), and Recursive Feature Elimination with Cross-Validation (RFECV), for screening variables in model construction.

### 2.5. Model construction

During the process of constructing and optimizing predictive models, this study utilized the scikit-learn library (version 1.3) in Python (version 3.9) and employed eleven machine learning algorithms: logistic regression, K-nearest neighbors (KNN), support vector machine, category boosting (CatBoost), decision tree, random forest, extreme gradient boosting (XGBoost), light gradient boosting machine (LightGBM), adaptive boosting (AdaBoost), multilayer perceptron (MLP), and Gaussian Naive Bayes (GaussianNB). Four different feature selection methods were integrated with these eleven algorithms for model training. Throughout the model training procedure, the learning curve was employed to examine the fitting efficacy of the model with respect to both the training set and the testing set (Figs. S3–S6, Supplemental Digital Content, https://links.lww.com/MD/P548). Model predictive performance was assessed using AUC and the average precision (AP) value, derived from the precision-recall (PR) curve. Furthermore, model accuracy, precision, recall, and F1 score were taken into account. Moreover, the calibration curve was harnessed to appraise the predictive calibration performance of the model, while the decision curve analysis (DCA) was utilized to gauge the net benefit as well as the optimal threshold of the model. To further validate the generalization capability of the model, the eICU dataset was adopted for external validation, aiming to conduct a comprehensive inspection of the model’s performance. For the purpose of comparing the predictive value of our proposed model with that of qSOFA, SOFA, and APACHE scores regarding the mortality rate of critically ill septic patients with hypoproteinemia, the AUC value was employed to evaluate the performance of our model against the existing scores. Subsequent to model evaluation, the best-performing model was identified, and the SHAP method was employed to elucidate in detail the impact of each feature on the prediction outcomes.

## 3. Results

### 3.1. Baseline features

Baseline features of the development cohort (MIMIC-IV dataset) and the external validation cohort (eICU dataset) are depicted in Table 1.

**Table 1:** The baseline tables for the MIMIC-IV dataset and the eICU dataset.

		MIMIC-IV			eICU		
Name	Levels	0 (N = 2767)	1 (N = 1091)	*P*	0 (N = 5236)	1 (N = 816)	*P*
Gender	Male	1579 (57.1%)	604 (55.4%)	.355	2716 (51.9%)	435 (53.3%)	.467
	Female	1188 (42.9%)	487 (44.6%)		2520 (48.1%)	381 (46.7%)	
Age	Median (IQR)	65.46 (54.48–76.92)	69.14 (59.20–79.23)	<.001	66.00 (54.50–76.00)	70.00 (60.00–79.00)	<.001
Race	White	2167 (78.3%)	782 (71.7%)	<.001	4390 (83.8%)	699 (85.7%)	.533
	Black	47 (1.7%)	16 (1.5%)		492 (9.4%)	72 (8.8%)	
	Asian	98 (3.5%)	28 (2.6%)		77 (1.5%)	10 (1.2%)	
	Other	455 (16.4%)	265 (24.3%)		277 (5.3%)	35 (4.3%)	
Infection	No	2183 (78.9%)	888 (81.4%)	.091	2633 (50.3%)	439 (53.8%)	.067
	Yes	584 (21.1%)	203 (18.6%)		2603 (49.7%)	377 (46.2%)	
NE	No	1045 (37.8%)	225 (20.6%)	<.001	4559 (87.1%)	630 (77.2%)	<.001
	Yes	1722 (62.2%)	866 (79.4%)		677 (12.9%)	186 (22.8%)	
CRRT	No	2680 (96.9%)	952 (87.3%)	<.001	5181 (98.9%)	805 (98.7%)	.562
	Yes	87 (3.1%)	139 (12.7%)		55 (1.1%)	11 (1.3%)	
Ventilation	No	1644 (59.4%)	489 (44.8%)	<.001	3030 (57.9%)	268 (32.8%)	<.001
	Yes	1123 (40.6%)	602 (55.2%)		2206 (42.1%)	548 (67.2%)	
Cerebrovascular	No	2482 (89.7%)	956 (87.6%)	.071	1870 (35.7%)	273 (33.5%)	.224
	Yes	285 (10.3%)	135 (12.4%)		3366 (64.3%)	543 (66.5%)	
Diabetes	No	2471 (89.3%)	982 (90%)	.557	3587 (68.5%)	617 (75.6%)	<.001
	Yes	296 (10.7%)	109 (10%)		1649 (31.5%)	199 (24.4%)	
Renal	No	2158 (78%)	794 (72.8%)	<.001	4312 (82.4%)	655 (80.3%)	.163
	Yes	609 (22%)	297 (27.2%)		924 (17.6%)	161 (19.7%)	
Pulmonary	No	2107 (76.1%)	795 (72.9%)	.037	3914 (74.8%)	589 (72.2%)	.128
	Yes	660 (23.9%)	296 (27.1%)		1322 (25.2%)	227 (27.8%)	
Aniongap	Median (IQR)	15.00 (13.00–18.00)	16.00 (13.00–20.00)	<.001	11.00 (8.00–14.00)	12.00 (9.00–15.00)	<.001
AST	Median (IQR)	43.00 (24.00–91.00)	55.00 (29.00–150.00)	<.001	31.00 (19.00–60.00)	51.00 (25.00–115.50)	<.001
ALP	Median (IQR)	93.00 (65.00–151.00)	104.00 (71.00–170.00)	<.001	84.00 (62.00–123.00)	93.00 (65.00–144.50)	<.001
ALT	Median (IQR)	31.00 (16.00–69.00)	32.00 (19.00–80.00)	.006	26.00 (16.00–47.00)	32.50 (18.00–74.50)	<.001
Lymphocytes	Median (IQR)	7.70 (4.00–12.70)	7.10 (4.00–12.00)	.332	7.80 (4.10–12.50)	6.00 (3.20–11.00)	<.001
Neutrophils	Median (IQR)	82.70 (74.00–88.20)	82.20 (71.75–88.30)	.112	82.80 (74.00–89.00)	83.70 (74.00–89.65)	.110
Monocytes	Median (IQR)	4.90 (3.00–7.30)	5.00 (3.00–8.00)	.354	6.00 (4.00–9.00)	5.05 (3.00–8.00)	<.001
Bicarbonate	Median (IQR)	21.00 (18.00–25.00)	21.00 (18.00–25.00)	.122	23.00 (19.00–26.00)	22.00 (18.00–25.00)	<.001
Bilirubin	Median (IQR)	0.80 (0.40–1.80)	1.00 (0.50–2.95)	<.001	7.90 (7.50–8.40)	7.90 (7.30–8.50)	.658
Calcium	Median (IQR)	8.00 (7.40–8.50)	8.10 (7.40–8.60)	.016	7.90 (7.50–8.40)	7.90 (7.30–8.50)	.658
Chloride	Median (IQR)	103.00 (99.00–108.00)	102.00 (97.00–107.00)	<.001	106.00 (101.00–110.00)	105.00 (100.00–110.00)	.010
Creatinine	Median (IQR)	1.20 (0.80–1.95)	1.40 (0.90–2.40)	<.001	1.20 (0.80–2.06)	1.53 (0.95–2.60)	<.001
Hematocrit	Median (IQR)	32.00 (27.80–36.40)	31.10 (26.40–35.80)	<.001	31.45 (27.30–35.80)	31.10 (26.85–36.00)	.730
Hemoglobin	Median (IQR)	10.50 (9.00–11.90)	10.00 (8.60–11.60)	<.001	10.30 (8.90–11.80)	10.20 (8.70–11.80)	.552
INR	Median (IQR)	1.40 (1.20–1.60)	1.50 (1.20–2.00)	<.001	1.35 (1.20–1.77)	1.46 (1.20–1.92)	<.001
Magnesium	Median (IQR)	1.80 (1.60–2.10)	1.90 (1.70–2.20)	<.001	1.80 (1.50–2.00)	1.90 (1.60–2.10)	<.001
MCH	Median (IQR)	30.10 (28.50–31.70)	30.50 (28.80–32.50)	<.001	29.70 (28.00–31.10)	29.80 (28.00–31.40)	.111
MCHC	Median (IQR)	32.70 (31.60–33.80)	32.60 (31.40–33.70)	.015	32.80 (31.90–33.70)	32.70 (31.70–33.70)	.094
MCV	Median (IQR)	92.00 (87.00–96.00)	94.00 (88.00–99.00)	<.001	90.00 (86.00–94.60)	90.70 (86.00–96.00)	.006
pco_2_	Median (IQR)	39.00 (33.00–45.00)	39.00 (32.00–47.00)	.394	37.00 (31.85–45.00)	37.40 (30.90–46.50)	.905
ph	Median (IQR)	7.37 (7.30–7.42)	7.35 (7.26–7.42)	<.001	7.37 (7.31–7.43)	7.34 (7.26–7.42)	<.001
Platelet	Median (IQR)	189.00 (122.50–269.00)	160.00 (91.00–252.50)	<.001	185.00 (129.00–258.00)	173.00 (100.00–256.00)	<.001
po_2_	Median (IQR)	75.00 (46.00–124.00)	80.00 (51.00–129.50)	.002	85.00 (67.10–117.00)	86.00 (66.70–130.00)	.261
Potassium	Median (IQR)	4.00 (3.60–4.50)	4.20 (3.70–4.80)	<.001	3.90 (3.50–4.40)	4.10 (3.60–4.60)	<.001
PT	Median (IQR)	15.00 (13.20–18.00)	16.00 (13.60–22.10)	<.001	16.00 (13.90–19.90)	17.00 (14.50–22.10)	<.001
PTT	Median (IQR)	31.50 (27.95–37.90)	34.50 (28.80–44.50)	<.001	35.20 (30.20–42.70)	37.00 (31.60–47.70)	<.001
Sodium	Median (IQR)	138.00 (134.00–141.00)	137.00 (133.00–141.00)	.027	138.00 (135.00–141.00)	138.00 (134.00–142.00)	.462
Ureanitrogen	Median (IQR)	24.00 (15.00–42.00)	31.00 (19.00–50.00)	<.001	25.00 (15.00–41.00)	35.00 (22.00–53.50)	<.001
WBC	Median (IQR)	12.50 (7.90–18.50)	11.90 (7.30–18.20)	.078	12.80 (8.60–18.10)	13.59 (9.00–20.35)	.008
RBC	Median (IQR)	3.52 (3.04–4.03)	3.33 (2.80–3.87)	<.001	3.51 (3.02–4.00)	3.43 (2.95–4.04)	.078
DBP	Median (IQR)	77.00 (66.00–89.00)	75.00 (65.00–88.00)	.016	61.00 (52.00–73.00)	59.00 (50.00–71.00)	.003
Glucose	Median (IQR)	130.00 (104.00–173.00)	132.00 (100.00–179.00)	.474	128.00 (102.00–170.00)	130.00 (103.00–174.00)	.715
Heart_rate	Median (IQR)	97.00 (82.00–113.00)	99.00 (84.00–114.00)	.058	97.00 (83.00–112.00)	100.00 (84.00–114.00)	.006
Lactate	Median (IQR)	1.90 (1.30–2.80)	2.20 (1.50–3.60)	<.001	1.60 (1.00–2.50)	2.21 (1.40–3.80)	<.001
RR	Median (IQR)	21.00 (17.00–25.00)	21.00 (18.00–26.00)	<.001	21.00 (17.00–25.00)	23.00 (18.00–28.00)	<.001
Sbp	Median (IQR)	64.00 (54.00–74.00)	62.00 (51.00–74.00)	.010	109.00 (95.00–128.00)	105.00 (91.00–124.00)	<.001
Temperature	Median (IQR)	98.40 (97.80–99.20)	98.10 (97.50–98.90)	<.001	98.40 (97.70–99.40)	98.20 (97.30–99.10)	<.001
UO	Median (IQR)	1480.00 (874.50–2295.00)	870.00 (343.50–1470.00)	<.001	275.00 (100.00–600.00)	100.00 (23.00–325.00)	<.001
GCS	Median (IQR)	14.00 (10.00–15.00)	10.00 (4.00–14.00)	<.001	14.00 (9.00–15.00)	5.00 (3.00–8.00)	<.001

Characteristics of acute pancreatitis patients with sepsis on admission: los_icu = duration of intensive care unit, infection = the first blood culture confirmed a bacterial infection, ventilation = early use of mechanical ventilation, cerebrovascular = presence of concomitant cardiovascular and cerebrovascular diseases, diabetes = presence of concomitant diabetes, renal disease = presence of concomitant renal disease, pulmonary = presence of concomitant chronic pulmonary disease.

ALP = alkaline phosphatase, ALT = alanine aminotransferase, AST = aspartate aminotransferase, CRRT = continuous renal replacement therapy, DBP = diastolic blood pressure, GCS = Glasgow Coma Scale, INR = international normalized ratio, MCH = mean corpuscular hemoglobin, MCHC = mean corpuscular hemoglobin concentration, MCV = mean corpuscular volume, NE =neutrophil, PT = prothrombin time, PTT = partial thromboplastin time, RBC, red blood cell, RR = respiratory rate, SBP = systolic blood pressure, UO = urine output in the first 24 hours, WBC = white blood cell.

### 3.2. Model Evaluation

**Figure 2.**
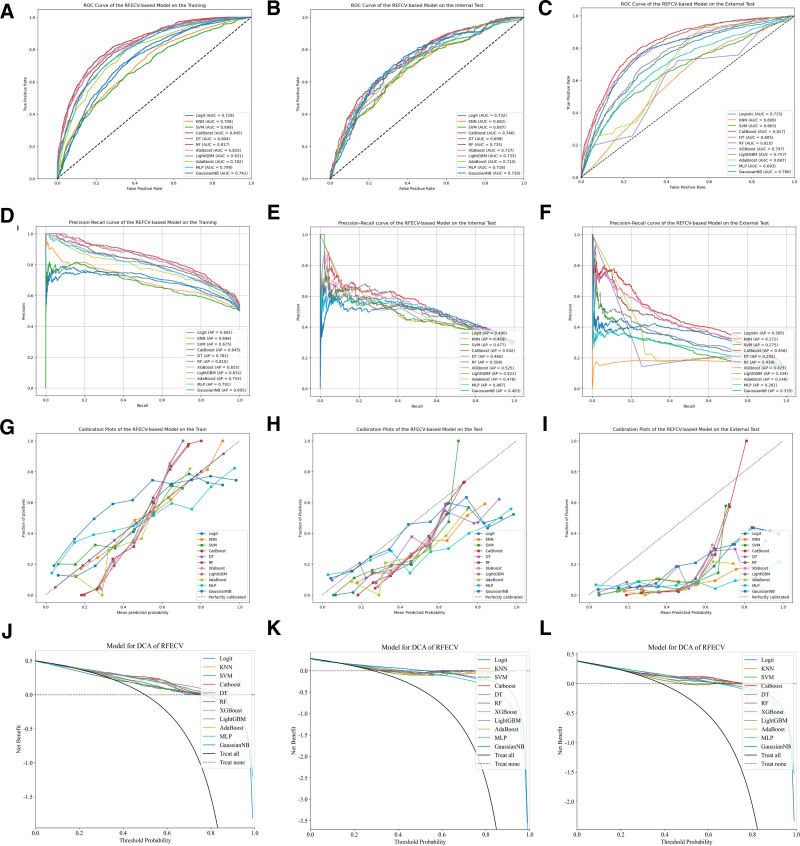
ROC and PR curves of the machine learning model constructed based on RFECV in the training set, internal validation set, and external validation set. PR = precision-recall, RFECV = recursive feature elimination with cross-validation.

**Figure 3.**
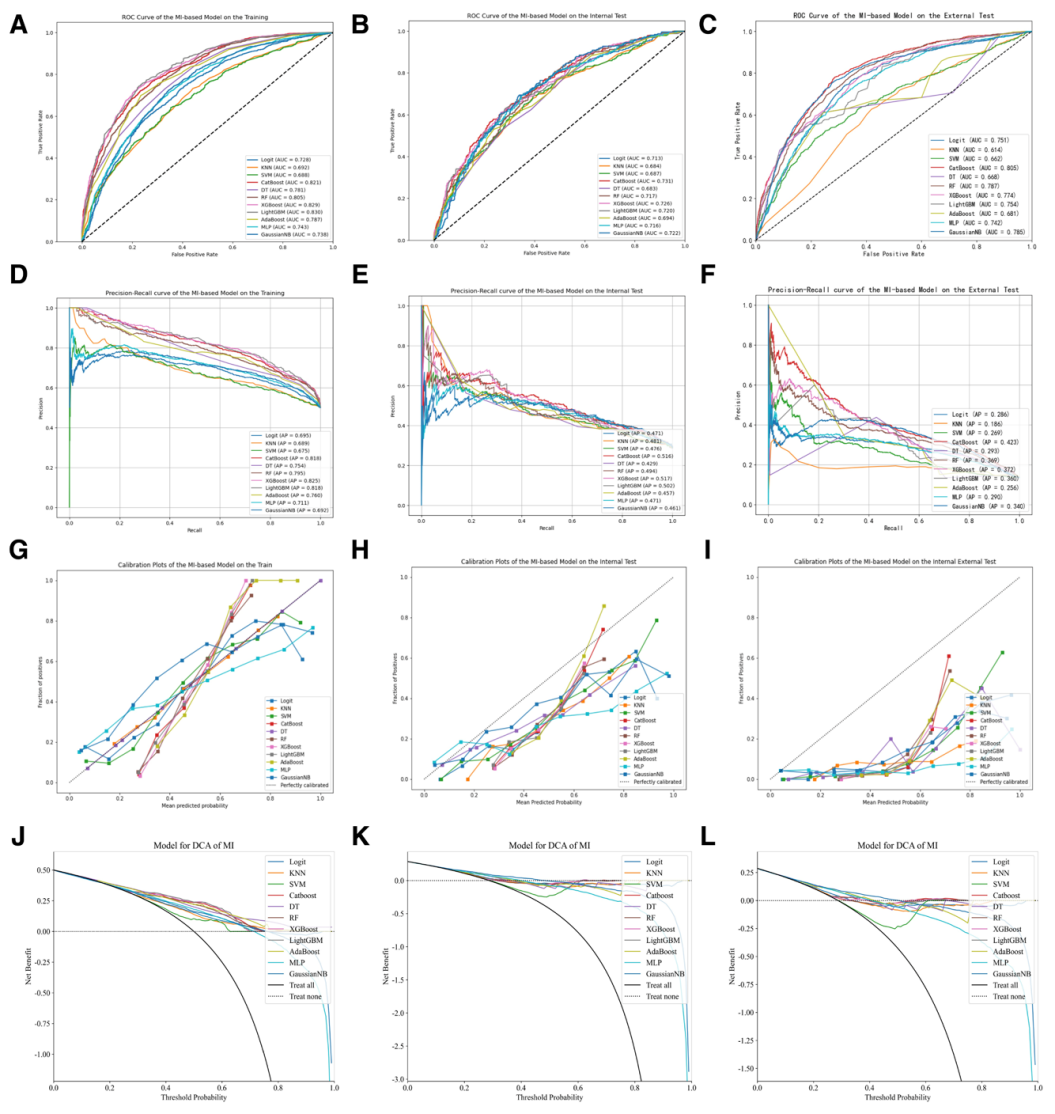
ROC and PR curves of the machine learning model constructed based on MI in the training set, internal validation set, and external validation set. MI = mutual information, PR = precision-recall.

**Figure 4.**
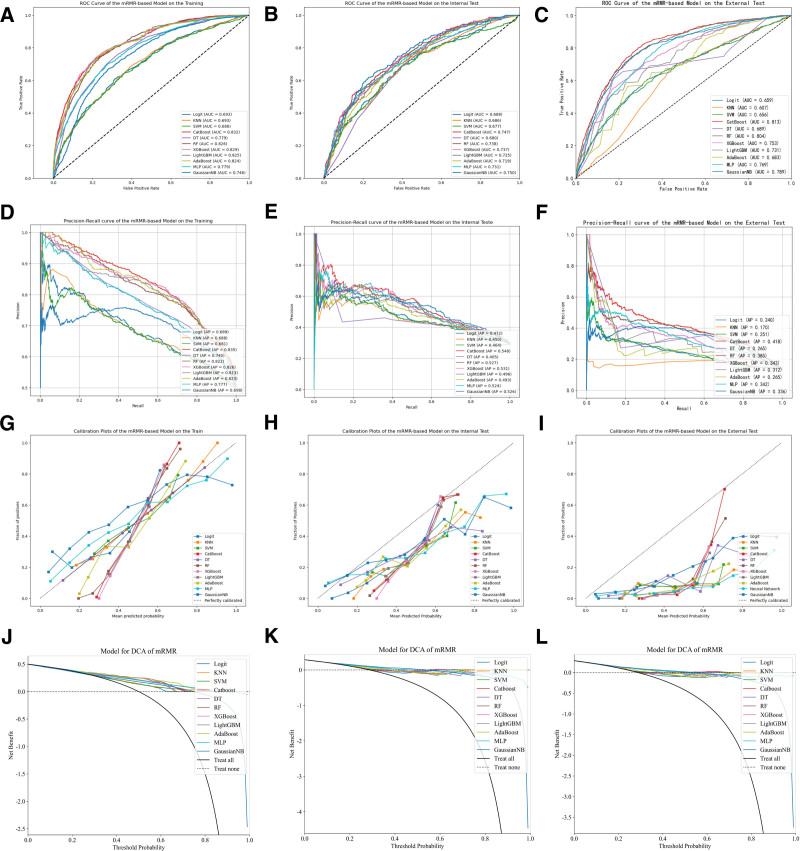
ROC and PR curves of the machine learning model constructed based on mRMR in the training set, internal validation set, and external validation set. mRMR = minimum redundancy maximum relevance, PR = precision-recall .

**Figure 5.**
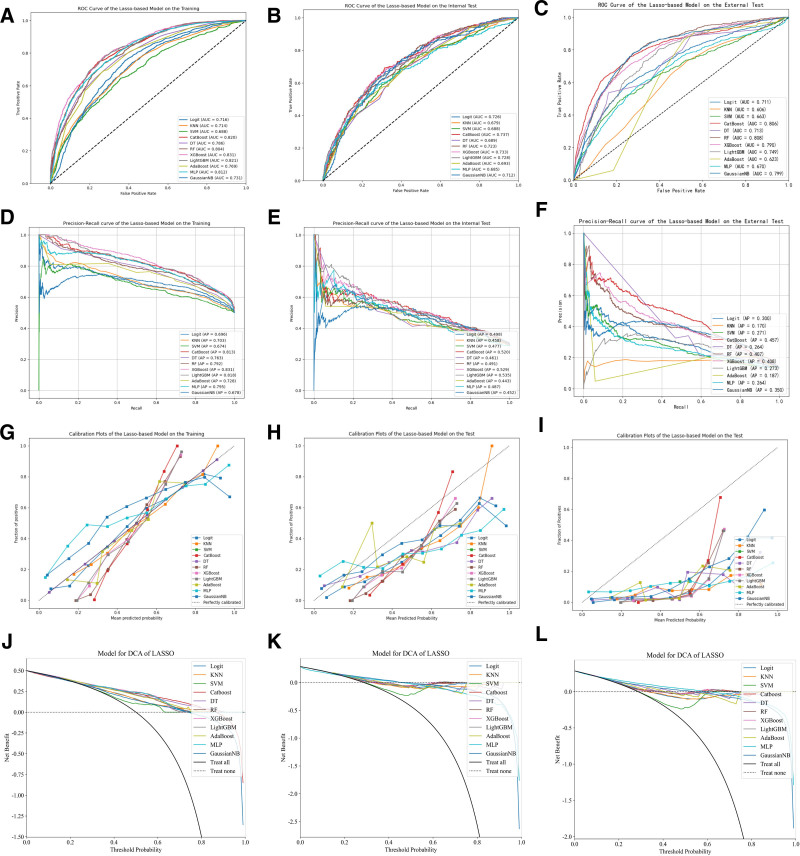
ROC and PR curves of the machine learning model constructed based on Lasso in the training set, internal validation set, and external validation set. PR = precision-recall.

In this study, we employed 4 feature selection methods in combination with eleven machine learning models, resulting in the construction of a total of forty-four distinct machine learning models. Due to data imbalance, we additionally utilized PR curves to evaluate the models.^[18]^ The PR curve, with Precision plotted on the horizontal axis and Recall on the vertical axis, provides a focused evaluation of positive instances’ performance. When addressing class imbalance issues, the PR curve is widely considered superior to the ROC curve, with a larger A *P*-value indicating better model performance. The remaining performance evaluation metrics included AUC, accuracy, precision, recall, and F1 score. The performance of each machine learning predictive model on the training set, internal validation set, and external validation set is presented in Table 2. The results revealed that, among all the models, the one constructed by combining RFECV and CatBoost manifested the optimal performance in predicting the mortality rate within the ICU for patients with early sepsis complicated by hypoproteinemia. Specifically, the AUC value of this model reached 0.845 in the training set, 0.746 in the testing set, and 0.827 in the external validation set, respectively, which collectively demonstrated its relatively outstanding predictive prowess. The ROC curves, PR curves, calibration curves, and DCA decision curves of all models built upon the variables screened through 4 feature engineering approaches are depicted in Figures 2 to 5. The PR curves demonstrated that the models constructed by the RFECV and CatBoost algorithms exhibited the most remarkable predictive performance when dealing with imbalanced data. The calibration curves illustrated that the CatBoost model boasted relatively precise calibration performance for the prediction outcomes. Meanwhile, the DCA decision curves also suggested that the CatBoost model possessed a relatively high net benefit threshold, further highlighting its superiority in practical application.

**Table 2:** Performance of models constructed using 4 feature selection methods and various machine learning algorithms on the training set, internal validation set, and external validation set.

		Training					Internal test					External test				
Feature	Model	AUC	Precision	Accuracy	Recall	F1	AUC	Precision	Accuracy	Recall	F1	AUC	Precision	Accuracy	Recall	F1
REFCV	LR	0.728	0.670	0.677	0.700	0.684	0.732	0.453	0.671	0.714	0.554	0.735	0.852	0.603	0.922	0.283
	KNN	0.708	0.634	0.642	0.672	0.652	0.682	0.409	0.628	0.669	0.507	0.606	0.813	0.528	0.924	0.246
	SVM	0.688	0.592	0.623	0.788	0.676	0.687	0.372	0.559	0.786	0.505	0.663	0.814	0.516	0.958	0.243
	Cat	**0.845**	0.741	0.764	0.811	0.774	0.746	0.447	0.667	0.791	0.543	0.827	0.874	0.661	0.952	0.317
	DT	0.804	0.705	0.725	0.775	0.738	0.698	0.419	0.639	0.675	0.517	0.605	0.775	0.510	0.757	0.233
	RF	0.817	0.728	0.744	0.780	0.753	0.735	0.438	0.658	0.687	0.535	0.810	0.870	0.691	0.915	0.343
	XGB	0.833	0.710	0.741	0.813	0.758	0.737	0.427	0.643	0.714	0.534	0.797	0.863	0.593	0.958	0.277
	GBM	0.831	0.702	0.740	0.834	0.763	0.733	0.414	0.629	0.711	0.523	0.757	0.863	0.587	0.961	0.274
	Ada	0.782	0.698	0.724	0.789	0.741	0.710	0.416	0.634	0.687	0.518	0.687	0.861	0.599	0.951	0.281
	MLP	0.799	0.721	0.726	0.738	0.729	0.718	0.459	0.679	0.675	0.546	0.693	0.833	0.601	0.843	0.283
	NB	0.741	0.744	0.647	0.450	0.561	0.730	0.524	0.726	0.497	0.51	0.780	0.855	0.724	0.659	0.437
MI	LR	0.728	0.666	0.663	0.652	0.659	0.713	0.445	0.668	0.651	0.529	0.751	0.855	0.595	0.939	0.278
	KNN	0.692	0.636	0.639	0.651	0.643	0.684	0.411	0.633	0.648	0.503	0.614	0.820	0.530	0.935	0.248
	SVM	0.688	0.524	0.544	0.658	0.677	0.687	0.311	0.571	0.562	0.471	0.662	0.848	0.507	0.925	0.240
	Cat	0.821	0.716	0.736	0.783	0.748	0.731	0.438	0.658	0.684	0.534	0.805	0.870	0.619	0.961	0.291
	DT	0.781	0.689	0.700	0.730	0.709	0.683	0.394	0.617	0.623	0.483	0.668	0.768	0.502	0.734	0.229
	RF	0.805	0.719	0.721	0.727	0.723	0.717	0.440	0.666	0.605	0.510	0.787	0.866	0.683	0.903	0.337
	XGB	0.829	0.717	0.740	0.791	0.752	0.726	0.437	0.659	0.663	0.527	0.774	0.867	0.603	0.963	0.282
	GBM	**0.830**	0.702	0.739	0.828	0.760	0.720	0.420	0.637	0.696	0.524	0.754	0.864	0.589	0.963	0.275
	Ada	0.787	0.698	0.719	0.772	0.733	0.694	0.398	0.619	0.642	0.491	0.681	0.833	0.582	0.877	0.272
	MLP	0.743	0.664	0.68	0.729	0.695	0.716	0.423	0.638	0.723	0.534	0.742	0.857	0.599	0.941	0.280
	NB	0.738	0.716	0.654	0.509	0.595	0.722	0.486	0.705	0.521	0.503	0.785	0.861	0.731	0.782	0.408
mRMR	LR	0.693	0.643	0.64	0.629	0.636	0.688	0.418	0.642	0.627	0.501	0.659	0.819	0.531	0.931	0.248
	KNN	0.693	0.636	0.644	0.674	0.654	0.686	0.408	0.627	0.672	0.508	0.607	0.818	0.527	0.939	0.246
	SVM	0.686	0.621	0.631	0.675	0.647	0.677	0.409	0.625	0.687	0.512	0.656	0.814	0.522	0.941	0.245
	Cat	**0.832**	0.732	0.746	0.776	0.754	0.747	0.445	0.666	0.675	0.537	0.813	0.870	0.640	0.951	0.304
	DT	0.779	0.699	0.715	0.757	0.727	0.680	0.414	0.634	0.669	0.512	0.689	0.798	0.539	0.801	0.249
	RF	0.826	0.743	0.748	0.759	0.751	0.738	0.458	0.680	0.620	0.527	0.804	0.866	0.694	0.890	0.349
	XGB	0.829	0.724	0.748	0.802	0.761	0.737	0.441	0.660	0.696	0.54	0.753	0.856	0.601	0.936	0.282
	GBM	0.825	0.720	0.747	0.810	0.762	0.725	0.434	0.655	0.678	0.529	0.731	0.855	0.613	0.924	0.288
	Ada	0.824	0.716	0.744	0.808	0.759	0.719	0.428	0.648	0.684	0.527	0.683	0.853	0.593	0.933	0.278
	MLP	0.779	0.732	0.707	0.653	0.690	0.731	0.481	0.699	0.599	0.534	0.769	0.864	0.658	0.919	0.317
	NB	0.746	0.731	0.678	0.563	0.636	0.750	0.525	0.730	0.602	0.561	0.789	0.859	0.734	0.691	0.443
Lasso	LR	0.716	0.640	0.660	0.731	0.683	0.726	0.415	0.627	0.738	0.531	0.711	0.835	0.548	0.938	0.256
	KNN	0.714	0.641	0.653	0.699	0.668	0.679	0.409	0.628	0.666	0.506	0.606	0.816	0.529	0.930	0.247
	SVM	0.688	0.525	0.545	0.658	0.678	0.688	0.311	0.376	0.670	0.471	0.663	0.848	0.507	0.995	0.24
	Cat	0.820	0.714	0.736	0.788	0.749	0.737	0.441	0.659	0.711	0.544	0.806	0.866	0.740	0.610	0.499
	DT	0.786	0.720	0.710	0.688	0.703	0.689	0.42	0.652	0.563	0.481	0.713	0.86	0.670	0.885	0.329
	RF	0.804	0.712	0.728	0.765	0.738	0.723	0.426	0.649	0.651	0.515	0.808	0.873	0.668	0.947	0.323
	XGB	**0.831**	0.717	0.747	0.815	0.763	0.733	0.431	0.649	0.708	0.536	0.790	0.866	0.594	0.964	0.278
	GBM	0.821	0.707	0.740	0.819	0.759	0.728	0.426	0.643	0.702	0.53	0.749	0.863	0.590	0.961	0.276
	Ada	0.769	0.700	0.703	0.708	0.704	0.693	0.414	0.642	0.593	0.488	0.623	0.866	0.673	0.912	0.329
	MLP	0.812	0.768	0.724	0.640	0.699	0.685	0.464	0.689	0.545	0.501	0.670	0.824	0.631	0.685	0.311
	NB	0.731	0.717	0.645	0.478	0.573	0.712	0.487	0.706	0.494	0.49	0.799	0.866	0.745	0.795	0.424

Bold indicates the model with the maximum AUC value for each combination of feature selection method and algorithm.

Ada = adaptive boosting, Cat = categorical boosting, DT = decision tree, GBM = gradient boosting machine, KNN = k-nearest neighbors, Lasso = least absolute shrinkage and selection operator, LR = logistic regression, MI = mutual information, MLP = multilayer perceptron, mRMR = minimum redundancy maximum relevance, NB = Gaussian Naive Bayes, REFCV = recursive feature elimination with cross-validation, RF = random forest, SVM = support vector machine, XGB = extreme gradient boosting .

### 3.3. Model interpretation

**Figure 6.**
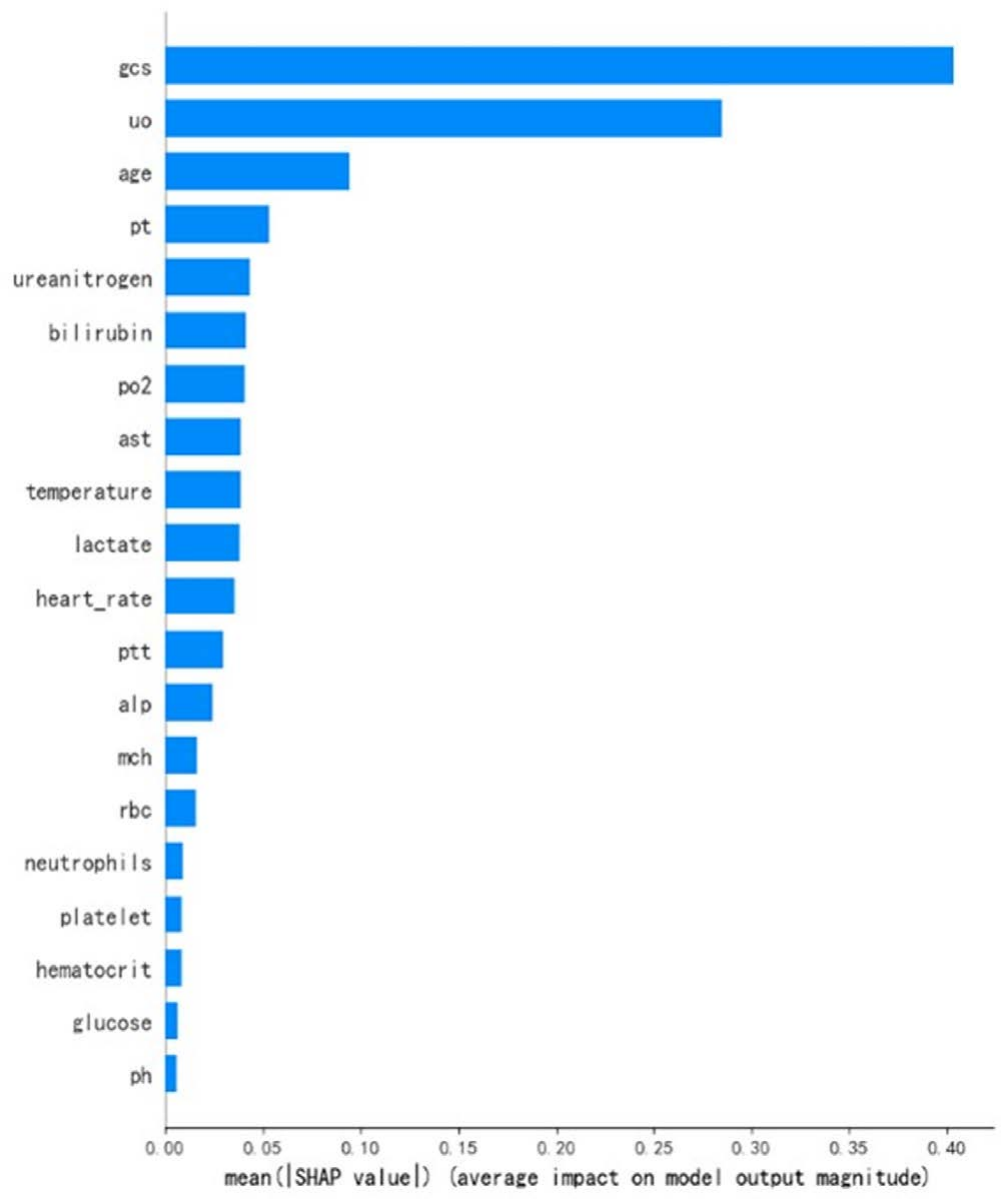
SHAP value feature importance ranking. SHAP = shapley additive explanations.

**Figure 7.**
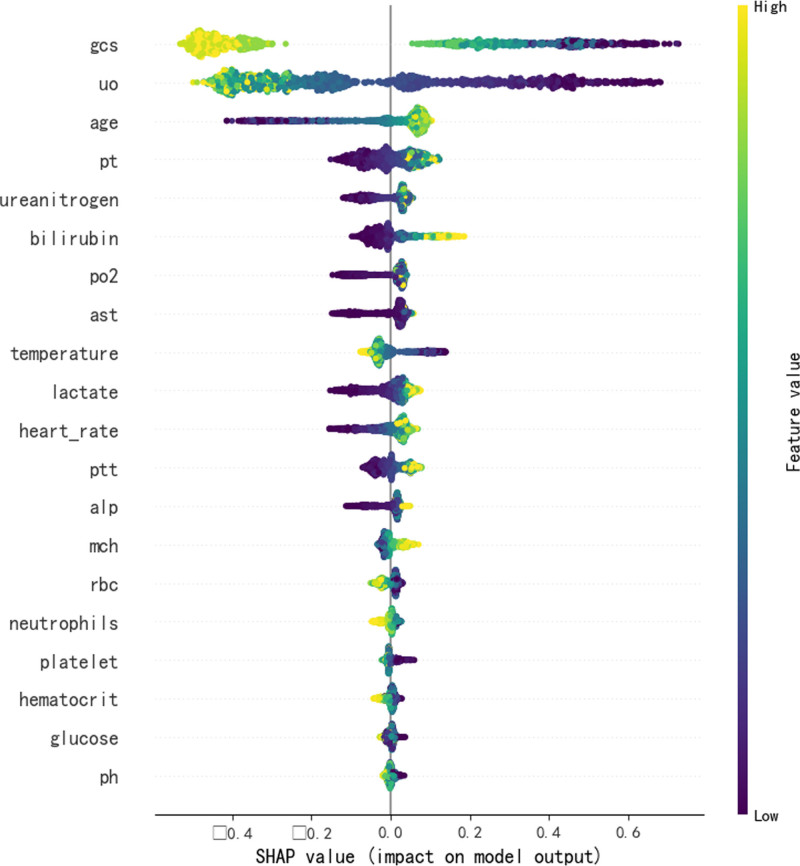
SHAP bee swarm plot. SHAP = shapley additive explanations.

**Figure 8.**
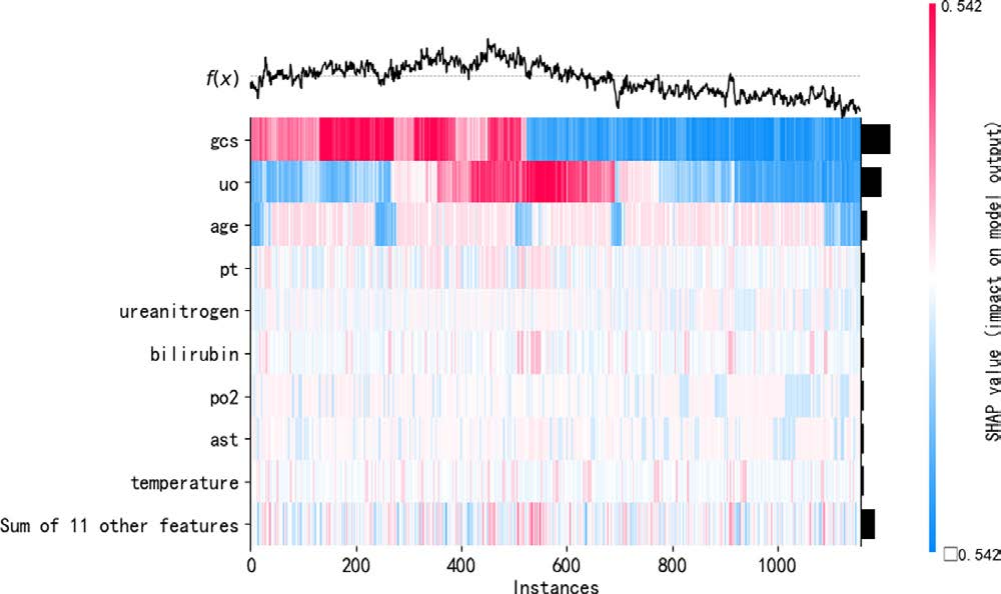
SHAP heatmap. SHAP = shapley additive explanations.

**Figure 9.**
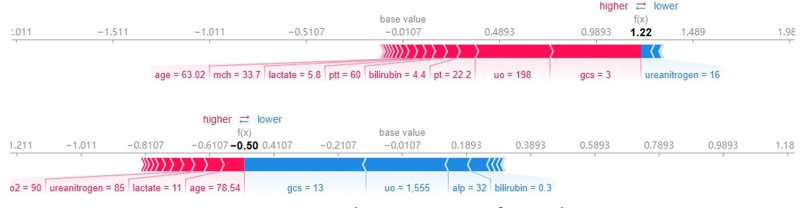
SHAP force plot. SHAP = shapley additive explanations.

To elucidate the decision-making process of the CatBoost model, we employed the SHAP algorithm to visually interpret the model’s predictions.^[19,20]^ SHAP combines the linearity of LIME’s explanations with the effectiveness of the Shapley value algorithm and is applicable to all machine learning models. It quantifies the contribution of each feature to the model’s predictions on both a global and local level. Examination of Figures 6 and 7 reveals the top 10 important features: GCS score, 24-hour urine output, age, prothrombin time, creatinine, bilirubin, partial pressure of oxygen, alanine aminotransferase, body temperature, and lactate. These features play a crucial role in predicting the prognosis of septic patients with hypoalbuminemia. Figure 7 demonstrates that low GCS score, oliguria, advanced age, prolonged prothrombin time, elevated creatinine, bilirubin, partial pressure of oxygen, alanine aminotransferase, hypothermia, and high lactate positively influence the model’s predictions, indicating an increased probability of mortality. This suggests that these factors act as risk factors for early mortality in septic patients with hypoalbuminemia admitted to the ICU. The SHAP heatmap in Figure 8 illustrates the contribution of these features to the prediction outcomes. Additionally, the SHAP force plot in Figure 9 delineates the contribution of each feature to individual prediction outcomes. Predictions are classified as positive when the final SHAP value for an individual prediction exceeds the base value; otherwise, they are classified as negative.

## 4. Model presentation and application

In order to facilitate the application of our research results to clinical physicians, relevant researchers, patients, and their families, we have developed a prognostic prediction system. This system can be evaluated on the following website: https://app12-3tvylgqzr5wd8t9w6jywdz.streamlit.app/

## 5. Discussion

In this study, hypoalbuminemia was defined as values below 3.5 g/dL. The MIMIC-IV database included a total of 3856 patients with sepsis and hypoalbuminemia, resulting in 1090 deaths and an overall in-hospital mortality rate of 28.3%. In our external validation cohort from the eICU, there were 6052 cases, with 816 deaths, leading to an overall in-hospital mortality rate of 13.5%. A substantial discrepancy in the mortality rates was observed between the 2 datasets (Table S1, Supplemental Digital Content, https://links.lww.com/MD/P547). This disparity could potentially be ascribed to the variances existing in multiple dimensions, encompassing patient characteristics, medical resources along with the corresponding interventions, and also data collection and its quality, between the MIMIC-IV database and the eICU database. Collectively, these factors contributed to the divergent situations regarding mortality rates and other aspects between the 2 databases. To improve the accuracy and generalization ability of the model, we employed 4 different feature selection methods for screening and compared the predictive performance of various machine learning models. The study results indicate that the model constructed with the combination of RFECV and CatBoost demonstrated the best performance. The generalization ability of this model was validated externally on the eICU database, showing good extrapolation. With a recall of 0.953, the CatBoost model can accurately identify early septic patients with hypoalbuminemia at high risk of death, thus aiding in predicting disease progression and assisting clinical physicians in timely intervention and bedside decision-making. Based on the DCA decision curve of our model, we identified the clinical decision threshold (Fig. S7, Supplemental Digital Content, https://links.lww.com/MD/P548). It was found that when the risk threshold fell within the range of 0.1 to 0.9, patients could attain the maximum net benefit. Simultaneously, in contrast to the qSOFA, SOFA, and APACHE scores that are currently employed for septic patients, the model developed by us exhibited a more outstanding performance (Fig. S8, Supplemental Digital Content, https://links.lww.com/MD/P548). Specifically, it was capable of predicting the mortality rate of patients with early sepsis complicated by hypoproteinemia in the ICU with a higher degree of precision, thereby providing more reliable references for clinical decision-making.

When septic patients are complicated by hypoalbuminemia, their prognosis tends to be poor.^[21]^ Research indicates that septic patients with hypoalbuminemia experience adverse outcomes such as prolonged hospitalization and higher mortality rates.^[22]^ Therefore, early prediction of mortality risk in septic patients with hypoalbuminemia, along with timely monitoring and intervention to correct hypoalbuminemia, is beneficial for improving patient outcomes and reducing mortality risk. Studies have suggested that transfusion of human albumin may reduce mortality risk in septic patients.^[23]^ Albumin administration can improve organ function in critically ill patients with hypoalbuminemia, restore fluid balance, and enhance tolerance to enteral nutrition. Building on this premise, we have developed a machine learning model specifically tailored to predict overall in-hospital mortality rates in early septic patients with hypoalbuminemia. Our predictive model facilitates clinical decision-making, enabling timely and personalized treatment for high-risk patients, thus enhancing efficacy and significantly reducing mortality risk. Its outstanding performance has been validated externally, demonstrating robust generalization ability and effective extrapolation to other datasets.

To enhance the precision of model construction, we gathered the initial laboratory test results and vital sign data of patients upon admission to the ICU. Additionally, we incorporated variables such as the early use of mechanical ventilation, infection status, administration of vasopressors and renal replacement therapy, as well as patient histories of cardiovascular disease, chronic kidney disease, diabetes, and chronic lung disease. A total of 57 variables were collected. The final CatBoost model, chosen RFECV, integrated easily accessible feature variables including GCSscore, 24-hour urine output, age, prothrombin time, creatinine, bilirubin, partial pressure of oxygen, alanine aminotransferase, body temperature, and lactate. These features function as predictors of mortality in septic patients with hypoalbuminemia, reflecting multi-organ dysfunction encompassing neurological disorders, renal and hepatic insufficiency, and respiratory failure.^[24]^ The risk of death in elderly patients often surpasses that of middle-aged and young individuals, consistent with prior investigations.^[25]^ Literature reports suggest that lower body temperature in sepsis signifies circulatory failure due to inadequate blood volume perfusion, culminating in hypothermia.^[26]^

This study utilized SHAP to elucidate the black-box nature of machine learning predictive models. The SHAP bee swarm plot illustrates the contribution of each feature to the model’s output. SHAP values reveal a close association between lower GCS scores, oliguria, elevated alanine aminotransferase, advanced age, dialysis treatment, body temperature, creatinine, and high mortality risk. The SHAP force plot demonstrates how factors such as GCS score, 24-hour urine output, aspartate aminotransferase, age, dialysis status, body temperature, alanine aminotransferase, and creatinine specifically influence individual prediction outcomes. SHAP provides an intuitive approach for machine learning predictive models to explain the specific impact of feature variables on prediction outcomes, enhancing clinical decision-makers’ understanding of model outputs and guiding clinicians in making clinical intervention decisions and personalized treatments.

In the present study, the developed model was established by leveraging diverse data sources, such as the outcomes of the initial laboratory examinations upon patients’ admission to the ICU, the relevant data regarding vital signs, as well as the early treatment circumstances following their admission to the ICU. Considering the distinctive features of the data underlying the model’s construction, upon comprehensive deliberation, we contend that the optimal time window for the application of this model lies within 24 hours after patients are admitted to the ICU, aiming to predict the mortality rate of patients with sepsis complicated by hypoproteinemia within 28 days. When applied within this specific time frame, the model can be maximally in line with its original design intention. By operating on the basis of the data conditions that closely resemble those during the model training process, it holds the promise of yielding relatively more precise and dependable results. Thereby, it can better facilitate clinical practice and support subsequent relevant analyses and decision-making processes.

This study presents several limitations. Firstly, owing to its retrospective design, there is a risk of selection bias. Secondly, the study is constrained by the content of the MIMIC-IV database, and the performance of our predictive model heavily depends on the accuracy of medical records. The diversity of patients’ conditions during hospitalization, along with the temporal variability in disease progression rates, could impact the model’s sensitivity. We conducted a limited analysis of the database, resulting in restricted inclusion of variables and the oversight of potential variables, potentially introducing bias. Furthermore, our model underwent external validation only in the eICU database, underscoring the need for multicenter data validation to bolster its generalizability. Moreover, on account of the restrictions arising from the study design and the limitations inherent in the database, our current research has hitherto failed to examine the performance of the model within different subgroups of the population. Lastly, while the model may aid in promptly identifying clinically high-risk scenarios, its capability to furnish additional insights into pathological physiological mechanisms that may jeopardize life could be constrained. Going forward, we plan to apply the model in real-world studies and conduct prospective validation to obtain feedback for further optimization, facilitating its continuous application in bedside clinical decision-making.

## 6. Conclusion

We have developed a machine learning model to predict the mortality rate of critically ill patients with early sepsis complicated by hypoalbuminemia in the ICU. The model has shown superior performance and achieved satisfactory results in both internal and external validation. This offers clinical decision-makers an accurate, objective, and efficient auxiliary tool to assist in disease prognosis. The SHAP method improves the interpretability of machine learning models, helping physicians better understand the reasons behind prediction outcomes. Moreover, the model demonstrates good clinical applicability as it does not rely on hard-to-obtain variables.

## Author contributions

**Conceptualization:** ZhanJin Wang, Ying Zhou, Zhan Wang.

**Data curation:** ZhanJin Wang, XuXia A.

**Formal analysis:** ZhanJin Wang, Lei Liu.

**Funding acquisition:** Lei Liu.

**Investigation:** FuYuan Li, ZhangTuo Xue.

**Methodology:** FuYuan Li, ZhangTuo Xue.

**Project administration:** JunJie Cai.

**Resources:** XuXia A, JunJie Cai.

**Software:** XuXia A.

**Supervision:** XuXia A, Kaihao Du.

**Validation:** Kaihao Du.

**Writing – original draft:** ZhanJin Wang, Zhan Wang.

**Writing – review & editing:** ZhanJin Wang, Ying Zhou, Zhan Wang.

## Supplementary Material


