# Supplementary material for: Prediction of the mortality rate in the intensive care unit for early sepsis patients with combined hypoalbuminemia based on machine learning
Source: Medicine (Baltimore). 2025 Aug 1;104(31):e43610. doi: 10.1097/MD.0000000000043610 (PMC12324020; doi:10.1097/MD.0000000000043610)
Supplement: Supplementary file 1 [file medi-104-e43610-s001.pdf]

Figure 1 shows the missing value data of the MIMIC database.

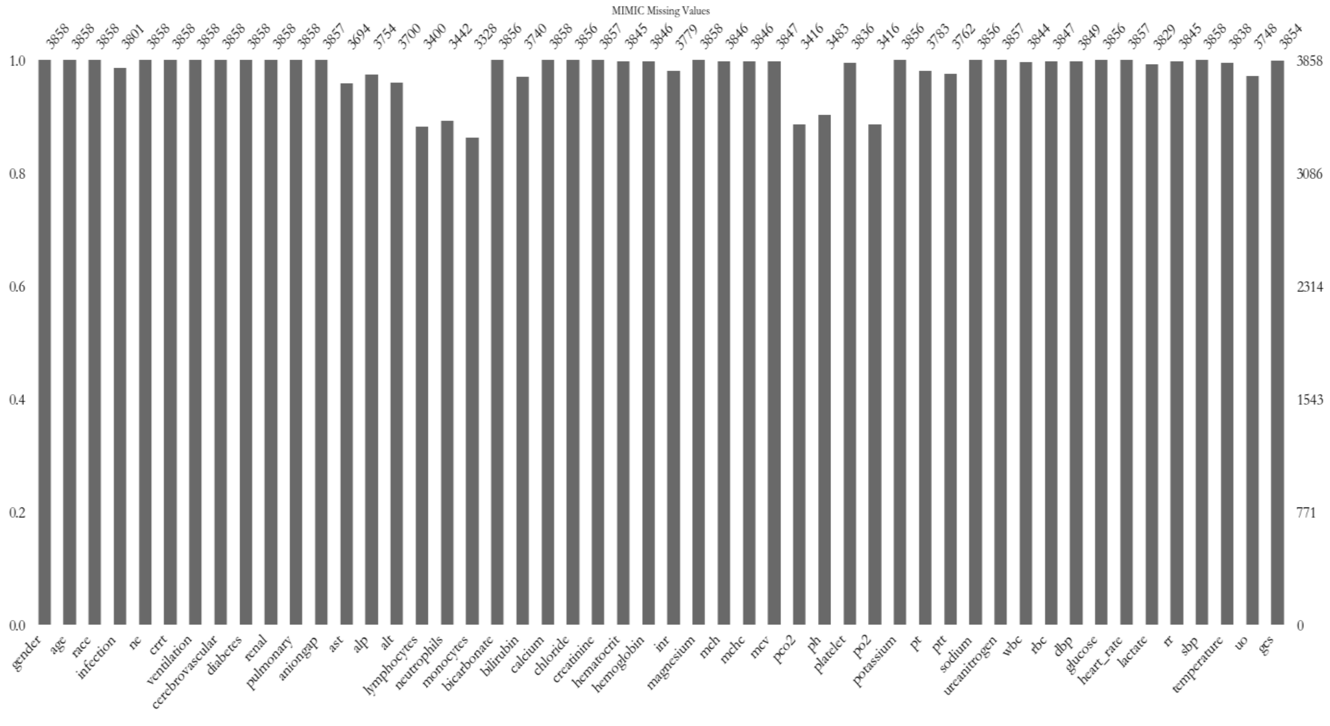


Figure 2 presents the missing value data of the eICU database.

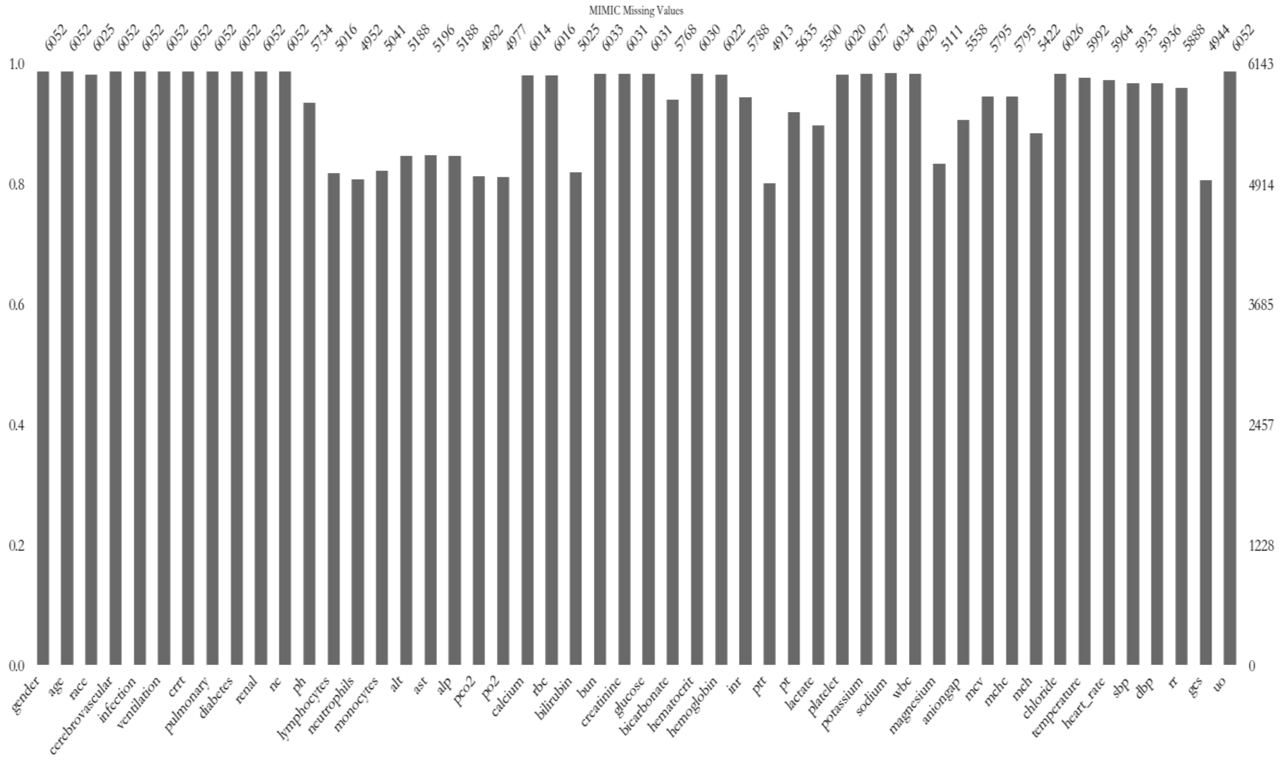

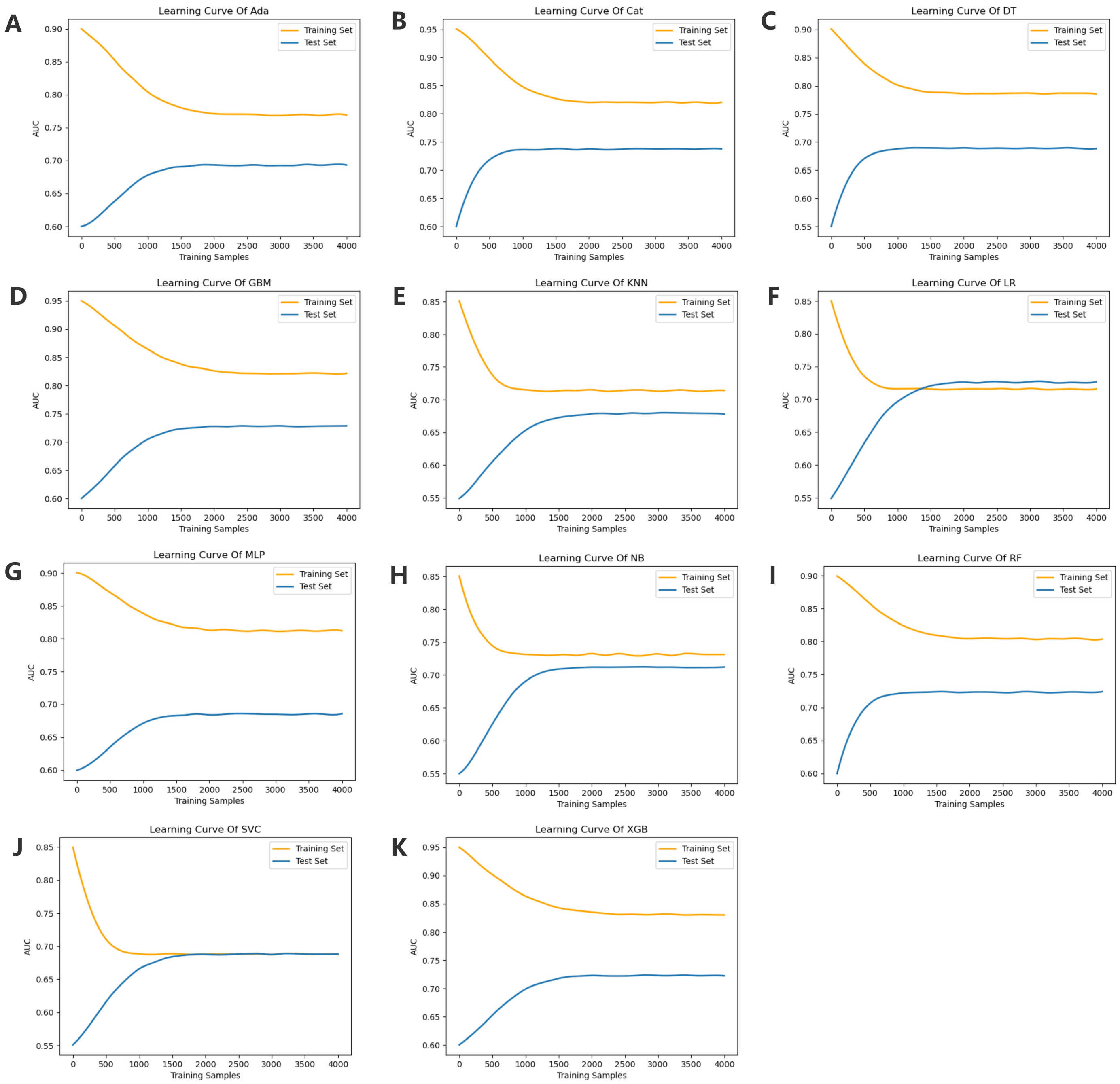


Ada: AdaBoost ;Cat: CatBoost ;DT: DecisionTree ;GBM:LightGBM ;KNN: K-Nearest Neighbors ;LR: Logistic Regression ;MLP: Multilayer Perceptron ;NB: Naive Bayes ;RF: Random Forest ;SVC:Support Vector Classifier ;XGB: XGBoost ;

The learning curve of the model constructed based on features screened by LASSO.

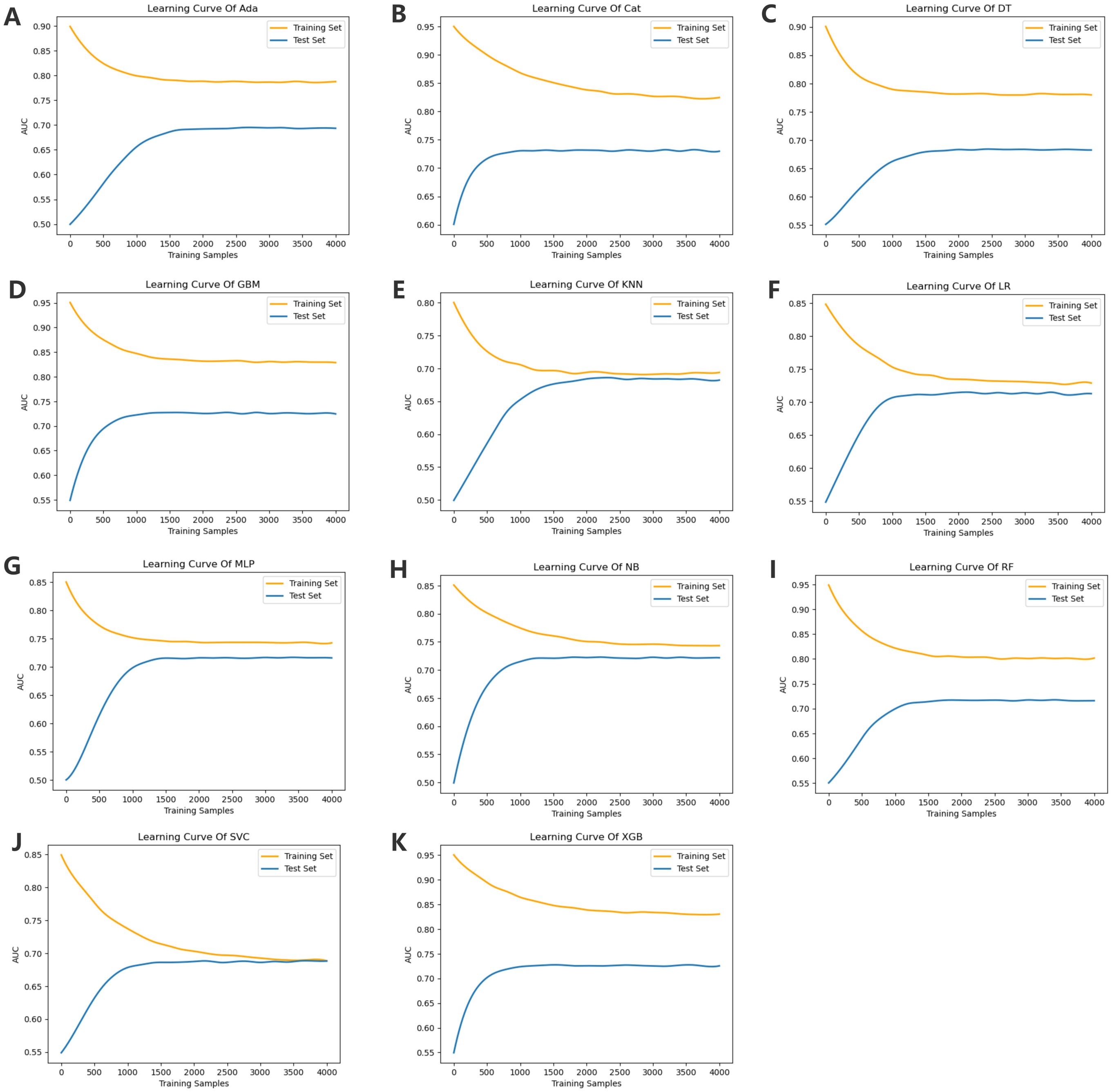


The learning curve of the model constructed based on features screened by MI.

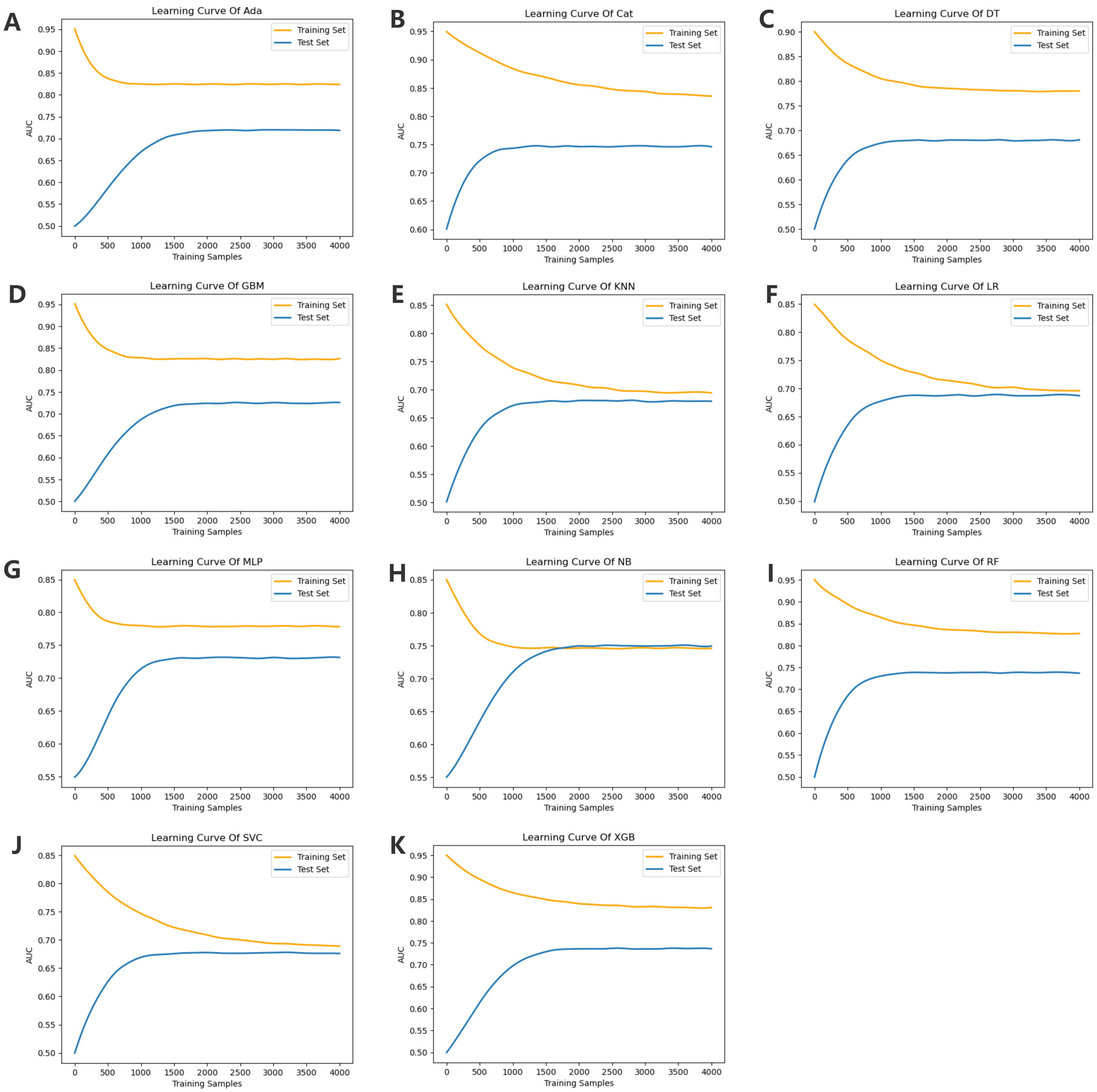


The learning curve of the model constructed based on features screened by mRMR.

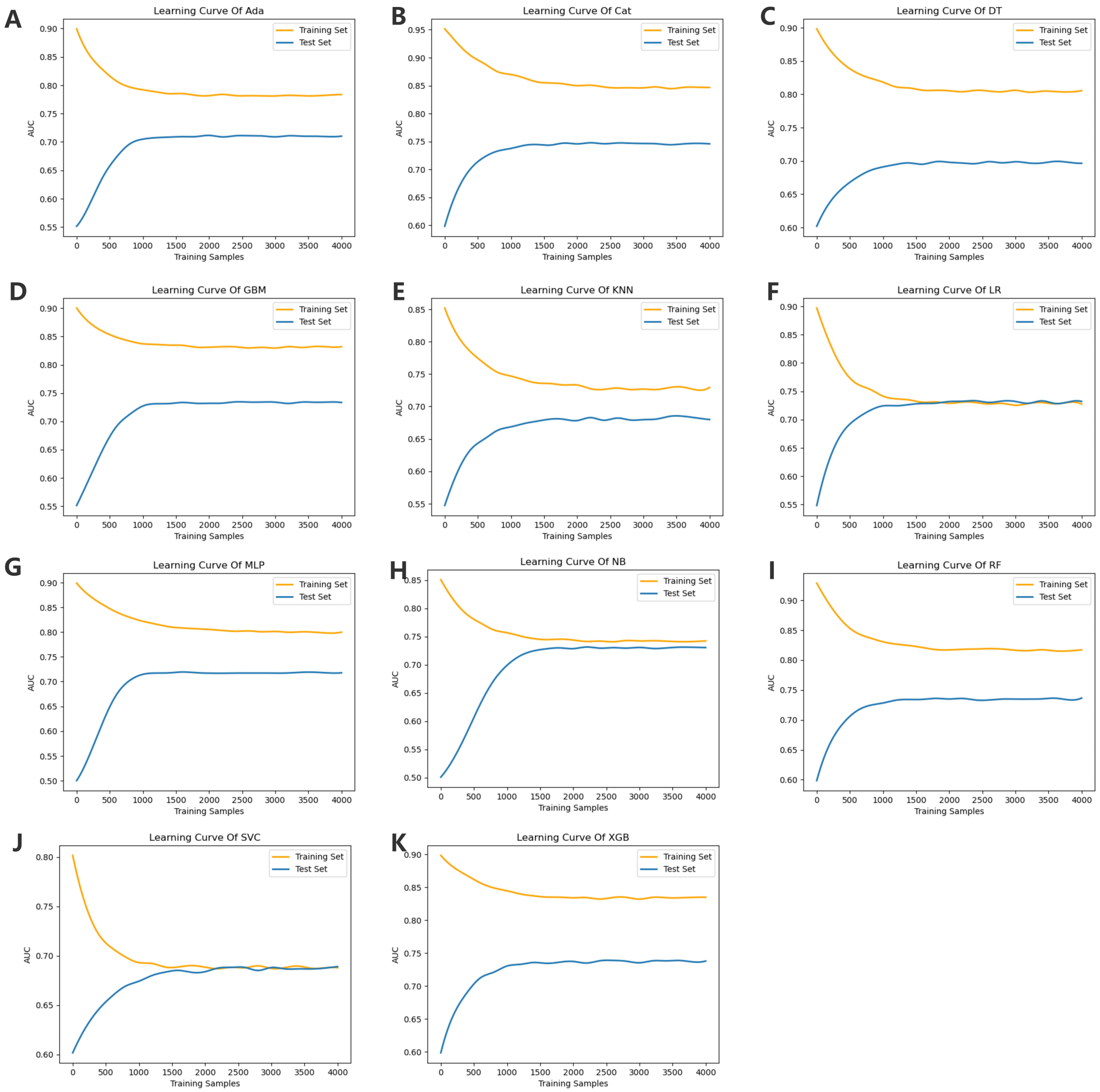


The learning curve of the model constructed based on features screened by RFECV.

Figure 7 shows the learning curve of the model constructed based on features screened by mRMR.

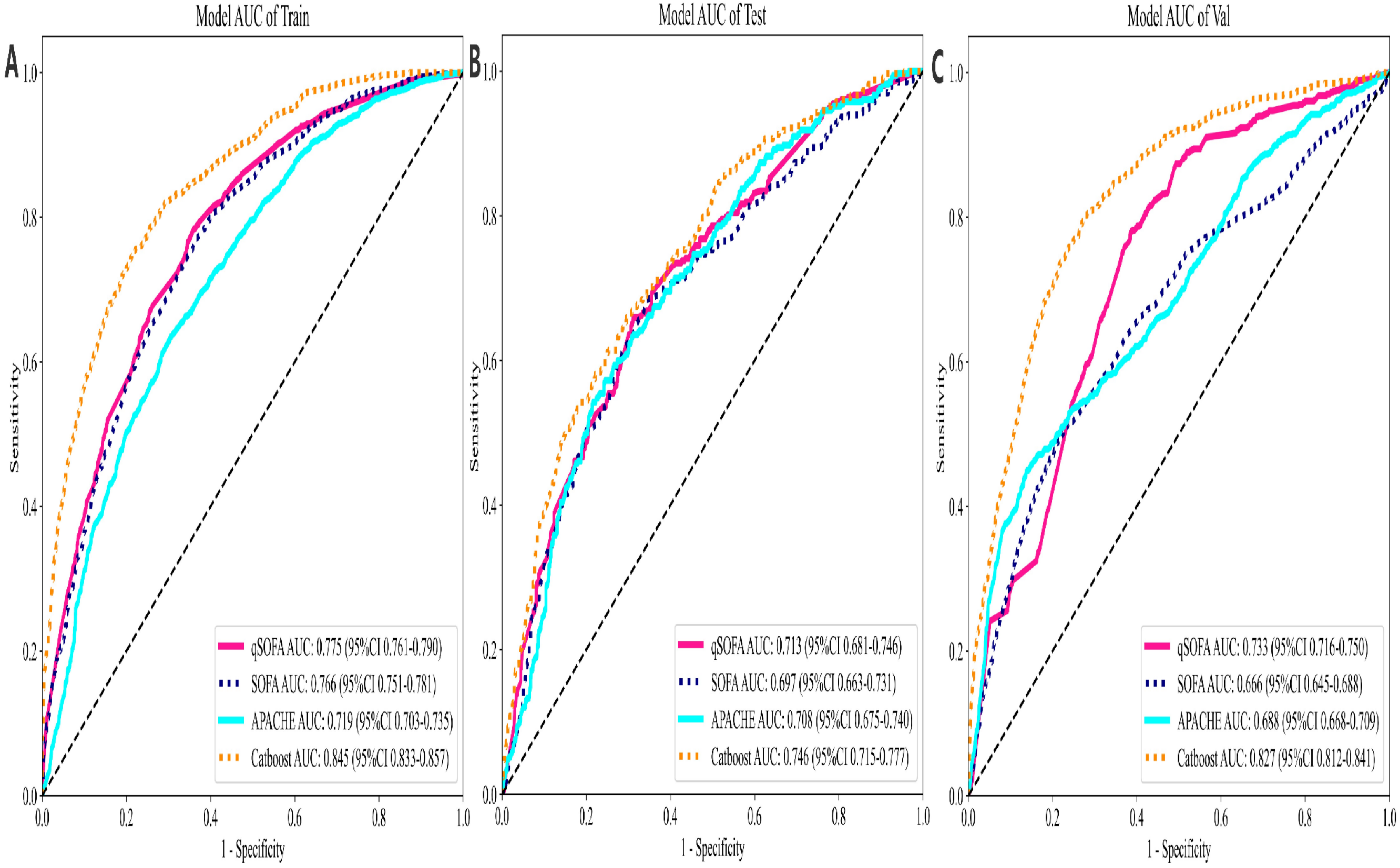

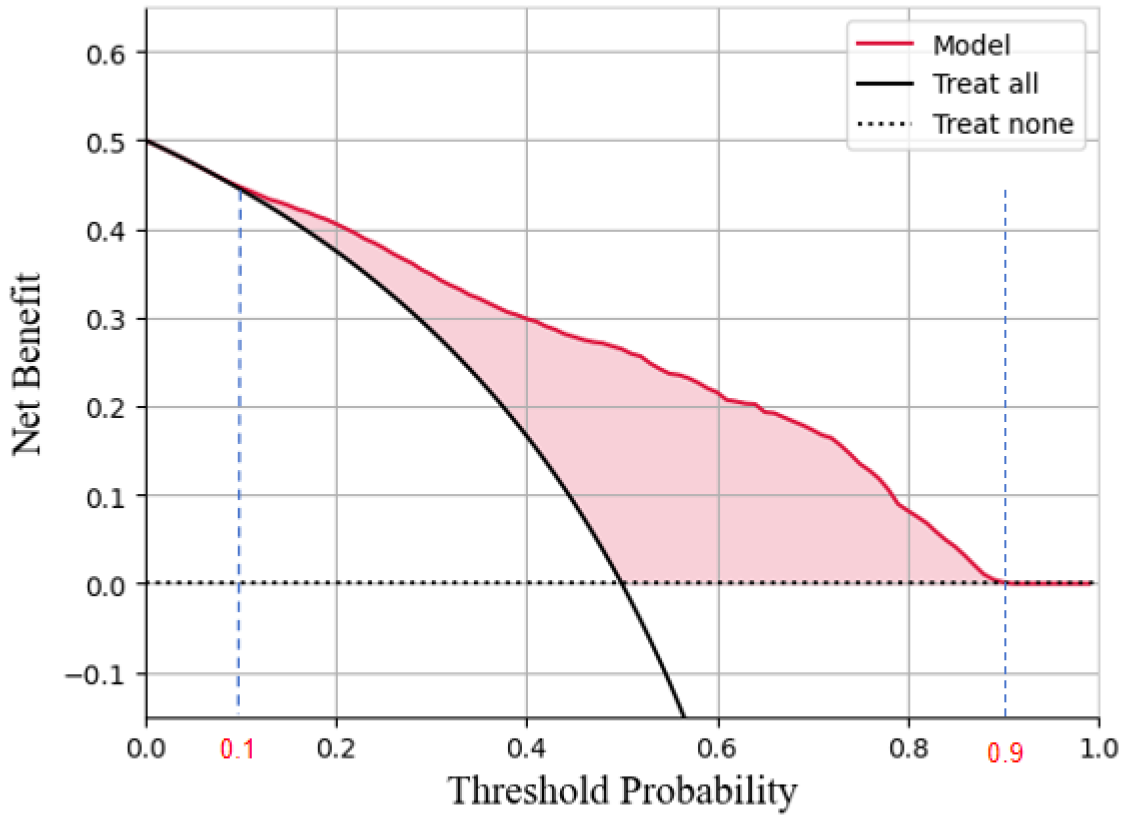


Figure 8 shows the learning curve of the model constructed based on features screened by RFECV.
