# Supplementary material for: Prediction of the mortality rate in the intensive care unit for early sepsis patients with combined hypoalbuminemia based on machine learning
Source: Medicine (Baltimore). 2025 Aug 1;104(31):e43610. doi: 10.1097/MD.0000000000043610 (PMC12324020; doi:10.1097/MD.0000000000043610)
Supplement: Supplementary file 2 [file medi-104-e43610-s002.docx]

表1. MIMIC-IV数据集和EICU数据集的基线表

| name | levels | MIMIC (N=3858) | eICU (N=6052) | p |
| --- | --- | --- | --- | --- |
| gender | Female | 2183 (56.6%) | 3150 (52%) | <.001 |
|  | Male | 1675 (43.4%) | 2902 (48%) |  |
| age | Median (IQR) | 66.8 (55.7 to 77.6) | 66.0 (55.0 to 76.0) | .061 |
| race | White | 2949 (76.4%) | 5089 (84.1%) | <.001 |
|  | Black | 63 (1.6%) | 564 (9.3%) |  |
|  | Asian | 126 (3.3%) | 87 (1.4%) |  |
|  | Other | 720 (18.7%) | 312 (5.2%) |  |
| infection | No | 3071 (79.6%) | 3072 (50.8%) | <.001 |
|  | Yes | 787 (20.4%) | 2980 (49.2%) |  |
| ne | No | 1270 (32.9%) | 5189 (85.7%) | <.001 |
|  | Yes | 2588 (67.1%) | 863 (14.3%) |  |
| crrt | No | 3632 (94.1%) | 5986 (98.9%) | <.001 |
|  | Yes | 226 (5.9%) | 66 (1.1%) |  |
| ventilation | No | 2133 (55.3%) | 3298 (54.5%) | .451 |
|  | Yes | 1725 (44.7%) | 2754 (45.5%) |  |
| cerebrovascular | No | 3438 (89.1%) | 2143 (35.4%) | <.001 |
|  | Yes | 420 (10.9%) | 3909 (64.6%) |  |
| diabetes | No | 3453 (89.5%) | 4204 (69.5%) | <.001 |
|  | Yes | 405 (10.5%) | 1848 (30.5%) |  |
| renal | No | 2952 (76.5%) | 4967 (82.1%) | <.001 |
|  | Yes | 906 (23.5%) | 1085 (17.9%) |  |
| pulmonary | No | 2902 (75.2%) | 4503 (74.4%) | .375 |
|  | Yes | 956 (24.8%) | 1549 (25.6%) |  |
| aniongap | Median (IQR) | 15.0 (13.0 to 19.0) | 11.0 (8.4 to 14.0) | <.001 |
| alt | Median (IQR) | 46.0 (25.0 to 104.0) | 27.0 (16.0 to 49.0) | <.001 |
| ast | Median (IQR) | 96.0 (66.0 to 157.0) | 33.0 (20.0 to 65.0) | <.001 |
| alp | Median (IQR) | 31.0 (17.0 to 71.0) | 85.0 (62.0 to 125.5) | <.001 |
| lymphocytes | Median (IQR) | 7.6 (4.0 to 12.6) | 7.0 (4.0 to 12.0) | .766 |
| neutrophils | Median (IQR) | 82.5 (73.3 to 88.2) | 83.0 (74.0 to 89.0) | .007 |
| monocytes | Median (IQR) | 5.0 (3.0 to 7.5) | 6.0 (4.0 to 9.0) | <.001 |
| bicarbonate | Median (IQR) | 21.0 (18.0 to 25.0) | 22.0 (19.0 to 26.0) | <.001 |
| bilirubin | Median (IQR) | 0.8 (0.4 to 2.1) | 0.6 (0.4 to 1.2) | <.001 |
| calcium | Median (IQR) | 8.0 (7.4 to 8.6) | 7.9 (7.4 to 8.4) | .010 |
| chloride | Median (IQR) | 103.0 (99.0 to 108.0) | 105.0 (101.0 to 110.0) | <.001 |
| creatinine | Median (IQR) | 1.2 (0.8 to 2.1) | 1.2 (0.8 to 2.1) | .998 |
| hematocrit | Median (IQR) | 31.8 (27.4 to 36.2) | 31.4 (27.2 to 35.8) | .006 |
| hemoglobin | Median (IQR) | 10.4 (8.9 to 11.9) | 10.3 (8.9 to 11.8) | .053 |
| inr | Median (IQR) | 1.4 (1.2 to 1.7) | 1.4 (1.2 to 1.8) | .041 |
| magnesium | Median (IQR) | 1.9 (1.6 to 2.1) | 1.8 (1.6 to 2.1) | <.001 |
| mch | Median (IQR) | 30.2 (28.6 to 31.9) | 29.7 (28.0 to 31.2) | <.001 |
| mchc | Median (IQR) | 32.7 (31.6 to 33.7) | 32.8 (31.9 to 33.7) | .005 |
| mcv | Median (IQR) | 92.0 (88.0 to 97.0) | 90.0 (86.0 to 94.8) | <.001 |
| pco2 | Median (IQR) | 39.0 (33.0 to 46.0) | 37.0 (31.7 to 45.0) | <.001 |
| ph | Median (IQR) | 7.4 (7.3 to 7.4) | 7.4 (7.3 to 7.4) | <.001 |
| platelet | Median (IQR) | 181.0 (115.0 to 266.0) | 184.0 (126.0 to 258.0) | .228 |
| po2 | Median (IQR) | 76.0 (47.0 to 126.0) | 85.0 (67.0 to 118.0) | <.001 |
| potassium | Median (IQR) | 4.1 (3.6 to 4.6) | 4.0 (3.5 to 4.5) | <.001 |
| pt | Median (IQR) | 15.2 (13.3 to 18.8) | 16.1 (14.0 to 20.3) | <.001 |
| ptt | Median (IQR) | 32.2 (28.1 to 39.5) | 35.5 (30.4 to 43.0) | <.001 |
| sodium | Median (IQR) | 138.0 (134.0 to 141.0) | 138.0 (135.0 to 141.0) | <.001 |
| ureanitrogen | Median (IQR) | 26.0 (16.0 to 44.0) | 26.0 (16.0 to 43.0) | .247 |
| wbc | Median (IQR) | 12.3 (7.7 to 18.4) | 12.9 (8.6 to 18.3) | <.001 |
| rbc | Median (IQR) | 3.5 (3.0 to 4.0) | 3.5 (3.0 to 4.0) | .032 |
| dbp | Median (IQR) | 76.0 (66.0 to 88.0) | 61.0 (51.0 to 73.0) | <.001 |
| glucose | Median (IQR) | 130.0 (103.0 to 175.0) | 128.0 (102.0 to 170.0) | .036 |
| heart_rate | Median (IQR) | 98.0 (83.0 to 114.0) | 97.0 (83.0 to 112.0) | .192 |
| lactate | Median (IQR) | 2.0 (1.4 to 3.0) | 1.6 (1.1 to 2.6) | <.001 |
| rr | Median (IQR) | 21.0 (17.0 to 26.0) | 21.0 (17.0 to 26.0) | .166 |
| sbp | Median (IQR) | 63.0 (53.0 to 74.0) | 109.0 (95.0 to 127.0) | <.001 |
| temperature | Median (IQR) | 98.3 (97.7 to 99.1) | 98.4 (97.7 to 99.3) | .004 |
| uo | Median (IQR) | 1300.0 (705.0 to 2100.0) | 250.0 (75.0 to 550.0) | <.001 |
| gcs | Median (IQR) | 13.0 (9.0 to 14.0) | 13.0 (7.0 to 15.0) | .090 |
| status | Alive | 2767 (71.7%) | 5236 (86.5%) | <.001 |
|  | Death | 1091 (28.3%) | 816 (13.5%) |  |
